# Replacement of GroEL in Escherichia coli by the Group II Chaperonin from the Archaeon Methanococcus maripaludis

**DOI:** 10.1128/JB.00317-16

**Published:** 2016-09-09

**Authors:** Riddhi Shah, Andrew T. Large, Astrid Ursinus, Bevan Lin, Preethy Gowrinathan, Jörg Martin, Peter A. Lund

**Affiliations:** a School of Biosciences, University of Birmingham, Edgbaston, Birmingham, United Kingdom; b Max Planck Institute for Developmental Biology, Tübingen, Germany; Princeton University

## Abstract

Chaperonins are required for correct folding of many proteins. They exist in two phylogenetic groups: group I, found in bacteria and eukaryotic organelles, and group II, found in archaea and eukaryotic cytoplasm. The two groups, while homologous, differ significantly in structure and mechanism. The evolution of group II chaperonins has been proposed to have been crucial in enabling the expansion of the proteome required for eukaryotic evolution. In an archaeal species that expresses both groups of chaperonins, client selection is determined by structural and biochemical properties rather than phylogenetic origin. It is thus predicted that group II chaperonins will be poor at replacing group I chaperonins. We have tested this hypothesis and report here that the group II chaperonin from Methanococcus maripaludis (Mm-cpn) can partially functionally replace GroEL, the group I chaperonin of Escherichia coli. Furthermore, we identify and characterize two single point mutations in Mm-cpn that have an enhanced ability to replace GroEL function, including one that allows E. coli growth after deletion of the *groEL* gene. The biochemical properties of the wild-type and mutant Mm-cpn proteins are reported. These data show that the two groups are not as functionally diverse as has been thought and provide a novel platform for genetic dissection of group II chaperonins.

**IMPORTANCE** The two phylogenetic groups of the essential and ubiquitous chaperonins diverged approximately 3.7 billion years ago. They have similar structures, with two rings of multiple subunits, and their major role is to assist protein folding. However, they differ with regard to the details of their structure, their cofactor requirements, and their reaction cycles. Despite this, we show here that a group II chaperonin from a methanogenic archaeon can partially substitute for the essential group I chaperonin GroEL in E. coli and that we can easily isolate mutant forms of this chaperonin with further improved functionality. This is the first demonstration that these two groups, despite the long time since they diverged, still overlap significantly in their functional properties.

## INTRODUCTION

Molecular chaperones are a group of diverse proteins, intensively studied over the last 3 decades, whose overall role is to maintain all components of the cellular proteome at their optimum level for activity by ensuring that they are fully folded (1–3). Chaperonins are a nearly ubiquitous subset of molecular chaperones, closely related by homology and active as double-ringed, cagelike oligomeric protein complexes made of 60-kDa monomers (4–6). They are found in all organisms except for a few mycoplasmas (7) and have been shown to be essential in bacteria, archaea, and eukaryotes (8–10). They are ATP-driven molecular machines that work by encapsulating unfolded or misfolded proteins that can then fold effectively in the secluded central cavity. Chaperonins are divided into two groups based on phylogeny: group I chaperonins (found principally in bacteria and eukaryotic organelles) and group II chaperonins (found in eukaryotic cytoplasm and in archaea, where they are generally referred to as thermosomes). The subunits of chaperonins from both groups share similar domain architecture and comprise three distinct domains: a flexible apical domain that harbors a client binding site, an equatorial domain that mediates ATP binding and hydrolysis, and an intermediate domain that relays allosteric signals between the apical and intermediate domain via two hinge regions. The subunits assemble into a large complex consisting of two rings of seven (group I) or eight or nine (group II) subunits, with the central cavities in the two rings being the likely site of protein folding during the ATP-driven protein-folding cycle (4–6, 11, 12).

Despite their homology, significant differences separate the two groups. For example, although many bacteria contain more than one chaperonin (13), in most cases studied they are homo-oligomeric, whereas group II chaperonins, with the exception of few archaeal chaperonins, are hetero-oligomeric. Second, the activity of a cochaperonin that caps the protein-folding cavity is a requirement for group I chaperonin function but does not apply to group II chaperonins, due to the presence of an additional helical protrusion from the protein's apical domain that provides this function (14, 15). Third, in group I chaperonins the subunits are staggered between the two rings, whereas in group II they are in register, and this appears to result in significant differences in their allosteric behavior (12, 15–18). Fourth, the surface charge distributions of the inner closed cavity show significant differences between the two groups (19). These differences have been attributed to the coevolution of chaperonins with their clients in respective host organisms. For example, the transition from homo-oligomeric chaperonin rings in group I to a mostly hetero-oligomeric distribution in group II has been suggested to have occurred in parallel with the evolution of complex proteins in higher organisms (20, 21), and the number of different subunits in organisms that encode group II chaperonins correlates strikingly with proteome size (22). Group II chaperonins have been reported to interact with faster-evolving proteins that have greater structural variety, in contrast to comparatively more conserved, slower-evolving clients for group I chaperonins (21, 23). ATPase allostery is altered from a concerted one in group I chaperonins to a sequential one in group II, and this has been suggested to mediate the ordered release of multidomain proteins bound specifically to selected subunits in group II chaperonins, in contrast to simultaneous release from all subunits in group I chaperonins (24, 25). The various surface properties of the chaperonin cage in group I and group II chaperonins have also been proposed to be associated with differential client specificity (19, 26).

GroEL from Escherichia coli and mitochondrial heat shock protein 60 (Hsp60) are unable to fold the major eukaryotic TRiC clients, actin and tubulin, *in vitro*, and it has also been reported that bacterial proteins that require GroEL assistance are unable to fold in the eukaryotic cytosol (27, 28). Moreover, in the archaeon Methanosarcina mazei, which encodes both group I and group II chaperonins, nonoverlapping sets of clients that exclusively require one of the two chaperonins were found, and some of these are predicted to be essential (23). All these studies imply that client optimization is a key driver of divergence between group I and group II chaperonins. However, further experimental evidence is still needed to support this hypothesis. Moreover, the precise nature of how structural differences between representatives of the two groups contribute to changes in their respective mechanisms of action is not fully resolved, one of the limitations being the high degree of structural complexity in most group II chaperonins and comparatively difficult *in vivo* analysis in archaeal and eukaryotic expression systems.

On the other hand, over 80% of the TRiC interactors have been shown to be recognized (though not folded) by GroEL (29), including the obligate TRiC clients actin and tubulin. This implies a relatively broad range of client specificity, probably reflecting common elements for client recognition between group I and group II chaperonins. The apparent client specificity of group II chaperonins thus may have more to do with their ability to fold specific clients rather than their ability to recognize them. Moreover, while the subunit-specific functionalization is complete in eukaryotic chaperonins, archaeal chaperonins do not show absolute subfunctionalization (30). Thus, group I and group II chaperonins may show some functional overlap, and this may be predicted to be more evident with archaeal thermosomes than with the more complex eukaryotic chaperonins.

Functional expression of a group II chaperonin in an organism that normally requires a group I chaperonin would help the understanding of the extent and basis of functional divergence between the two groups and would also allow genetic dissection of the properties of group II chaperonins in a simple host. We have therefore investigated whether an archaeal chaperonin can replace GroEL function in E. coli, using the chaperonin from the mesophilic archaeon Methanococcus maripaludis, which we designate Mm-cpn. It was chosen for the following reasons: (i) Mm-cpn is homo-oligomeric, which as well as facilitating recombinant expression and mutagenesis in bacterial hosts means that it represents the simplest form of thermosome; (ii) the optimum temperature of growth for the parent organism is 35°C to 40°C, so the chaperonin is expected to exhibit functionality at roughly the same temperature optimum as that of E. coli; and (iii) this chaperonin has already been expressed in E. coli and characterized for ATPase and *in vitro* folding properties, and its structure has been solved (31–34).

## MATERIALS AND METHODS

### Strains and plasmids.

The strain TAB21 was derived from E. coli MGM100 (31) and contains the *groE* operon under the control of the arabinose- and glucose-regulated p*BAD* promoter in a BL21 background. AI90 is a strain in which the chromosomal copy of *groEL* has been replaced by a kanamycin resistance cassette. Growth is maintained by *ptrc*-regulated *groEL* on an expression plasmid that also carries a *sacB* gene to enable selection for cells that have lost this plasmid after introducing plasmids expressing proteins whose ability to complement is to be assayed (32, 33). Vectors pET21b(+) (Novagen) and pACYC184 were used for constructing all the plasmids used for complementation assays in TAB21 and AI90, respectively.

### Plate assay for complementation of GroEL function.

TAB21 cells containing test plasmids were grown overnight at 37°C with 0.2% arabinose and then diluted and grown for 2 h in LB medium containing 0.2% glucose. Cells equivalent to an optical density at 600 nm (OD_600_) of 1 were harvested and washed three times using LB broth. Tenfold dilutions of the washed cells were spotted onto LB agar plates containing 0.2% arabinose or 0.2% glucose with or without isopropyl-β-d-thiogalactopyranoside (IPTG; 1 mM) and incubated at 26°C, 30°C, or 37°C. AI90 cells were transformed with appropriate plasmids and grown on LB agar plates containing sucrose with or without IPTG. The plates were incubated at 26°C, 30°C, or 37°C and scored for growth.

### Random mutagenesis using XL-1 mutator strain.

The pET21-*Mm-cpn* plasmid was transformed into XL-1 Red strain (Agilent Technologies), and cells were plated onto LB agar containing ampicillin. All the colonies obtained were mixed together by resuspending in 500 μl LB on the plate and were cultured overnight at 37°C in 100 ml of LB containing ampicillin. For a higher mutation rate, these cultures were subcultured and grown overnight for two cycles. After this, plasmids were isolated, transformed into TAB21, and grown on plates containing LB agar with kanamycin, ampicillin, 0.2% glucose, and 1 mM IPTG. Plates were screened visually for faster-growing colonies.

### Purification of mutant Mm-cpn.

Purification was carried out essentially as described previously (34, 35) with modifications as described below. Cultures of E. coli BL21(DE3) (Novagen) cells carrying the pET plasmid constructs of Mm-cpn mutants were grown from an overnight culture in LB medium at 37°C until an OD_600_ of 0.4 was achieved. Protein expression was induced by addition of 1 mM IPTG, and the cultures were further grown for 4 h. Cells were harvested by centrifugation, and pellets were resuspended in lysis buffer (30 mM Tris-HCl [pH 7.5], 50 mM NaCl, 5 mM MgCl_2_, 15% ethanol [vol/vol], 1 mM phenylmethylsulfonyl fluoride [PMSF], 1 mM protease inhibitor cocktail [Roche]). After lysis of the cells using a French press, the lysate was centrifuged twice at 25,000 × *g* and filtered through a 0.2-μm filter. The clear supernatant was applied to a Q high-performance (QHP) anion exchange column preequilibrated with column buffer A (30 mM Tris-HCl [pH 7.5], 5 mM NaCl, 5 mM MgCl_2_, 1 mM dithiothreitol [DTT]). Elution was achieved with a linear gradient of 0 to 50% of column buffer B (30 mM Tris-HCl [pH 7.5], 1 mM NaCl, 5 mM MgCl_2_, 1 mM DTT). Fractions were analyzed by SDS-PAGE and native PAGE for the presence of assembled Mm-cpn. The fractions containing Mm-cpn were pooled and diluted 4-fold into column buffer A and applied to a MonoQ 16/10 anion exchange column (GE). Elution was achieved as described above, and the relevant fractions were identified by running on 12% SDS-PAGE. These fractions were pooled and precipitated with 70% ammonium sulfate, resuspended in column C buffer (30 mM Tris-HCl [pH 7.5], 100 mM KCl, 5 mM MgCl_2_, 1 mM DTT), and applied to an S300 HiPrep 26/60 column (GE) preequilibrated with buffer C. After analysis by SDS-PAGE and native PAGE, relevant fractions were pooled and concentrated up to 10 mg/ml using Amicon Ultra-15 centrifuge devices. The pure preparations were supplemented with 10% glycerol and stored at −70°C.

### Enzymatic activity assays.

For the citrate synthase (CS) aggregation assay, 200 nM subunits (100 nM dimer) porcine heart citrate synthase (Sigma) was mixed in 1 ml of CS assay buffer (30 mM morpholinepropanesulfonic acid [MOPS]-KOH [pH 7.5], 300 mM KCl, 5 mM MgCl_2_) with Mm-cpn proteins in a 1:1 molar ratio (i.e., 200 nM Mm-cpn hexadecameric complex). The reaction mixture was incubated in a preheated water bath at 50°C. The extent of aggregation was followed by measuring the light scattering at 320 nm over a period of 30 min. ATP hydrolysis activity was determined by measuring liberated P_i_ in a malachite green assay as described previously (35, 36). Briefly, ATP and Mm-cpn protein were mixed (final concentrations, 1 mM and 5 μM complex, respectively) in an ATPase assay buffer (30 mM Tris-HCl [pH 7.5], 300 mM KCl, 5 mM MgCl_2_, and 1 mM DTT) and incubated at 37°C for 15 min. The reaction was stopped by addition of 10 mM EDTA followed by colorimetric detection (640 nm). Rhodanese reactivation was done as described previously (34). Proteinase K sensitivity was measured by incubating purified Mm-cpn proteins and its mutants at a concentration of 0.8125 μM complex in 50 μl of assay buffer (30 mM MOPS-KOH [pH 7.5], 300 mM KCl, 5 mM MgCl_2_, 5 mM MgCl_2_, and 1 mM DTT). The reaction was started by addition of ATP or ADP to a final concentration of 1 mM. When metal fluoride complexes were to be generated, AlFx [5 mM Al(NO3)_3_ and 30 mM KF] was added to the mixture before the addition of ATP or ADP. Next, 1 μg/ml proteinase K was added, and the samples were incubated for 15 min at 30°C. PMSF (5 mM; 100% [vol/vol] in ethanol) was added to inhibit protease activity, and the samples were immediately mixed with SDS loading dye to be analyzed by 12% SDS-PAGE.

### Western blotting.

Following the separation of proteins by SDS-PAGE, electrotransfer to polyvinylidene difluoride (PVDF) membranes (Immobilon-P), and overnight blocking with nonfat dried milk, membranes were probed with a 1:1,000 dilution of a rabbit polyclonal antiserum raised against the thermosome of Thermoplasma acidophilum, generously provided by Gundula Bosch. Bound antibody was detected using the ECL Plus kit (Amersham).

## RESULTS

### Group II chaperonin from the archaeon Methanococcus maripaludis can sustain growth of E. coli cells depleted for GroES and GroEL.

**FIG 1:**
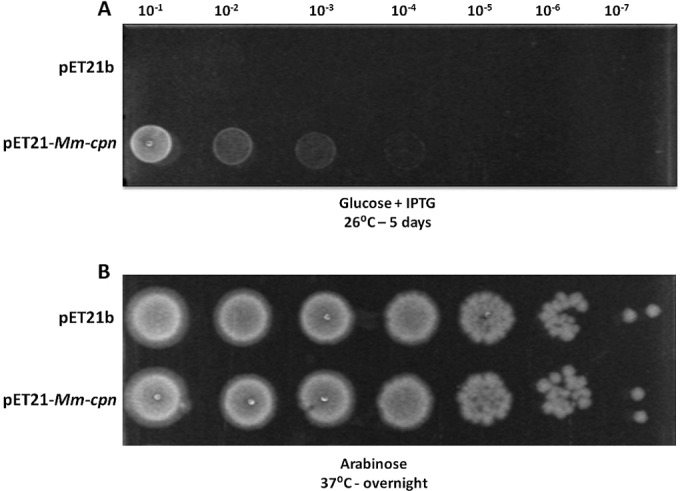
M. maripaludis chaperonin Mm-cpn can partially rescue growth of E. coli TAB21 under GroE-limiting conditions. TAB21 cells containing plasmids pET21 and pET21-*Mm-cpn* grown on 0.2% glucose (*groE* repression) and 1 mM IPTG (Mm-cpn induction) at 26°C (A) and on arabinose (*groE* induction) at 37°C (B).

E. coli strain TAB21 is a derivative of strain BL21 in which the chromosomal *groE* promoter has been replaced by the pBAD promoter (37). Repression of the pBAD promoter function in TAB21 cells leads to an inability to grow on glucose-containing medium, as GroES and GroEL are essential for survival and are depleted once this promoter has been repressed, largely by dilution as cells divide. Complementation by another chaperonin will occur only if this chaperonin can functionally replace GroEL and GroES. We used this strain to test the ability of Mm-cpn to act as a functional chaperonin in E. coli. TAB21 was transformed with a pET21-derived plasmid expressing the Mm-cpn gene under the control of an IPTG-inducible T7 promoter, and expression and assembly of the Mm-cpn protein was confirmed when the promoter was induced. To test the ability of Mm-cpn to complement for loss of GroES and GroEL function, TAB21 cells expressing Mm-cpn were grown in the presence of arabinose overnight and then transferred to media containing glucose. Dilutions of these cultures were spotted onto plates containing glucose or arabinose, and these were then incubated at 26°C, 30°C, and 37°C. Cells expressing Mm-cpn showed growth and formed small colonies on plates containing glucose after approximately 5 days of incubation at 26°C and 30°C (Fig. 1A), showing that Mm-cpn can at least partially overcome the lack of growth caused by depletion of GroES and GroEL. No growth was observed at 37°C unless arabinose replaced glucose in the medium (Fig. 1B).

### Selection of Mm-cpn variants showing enhanced ability to replace GroES and GroEL in E. coli.

**FIG 2:**
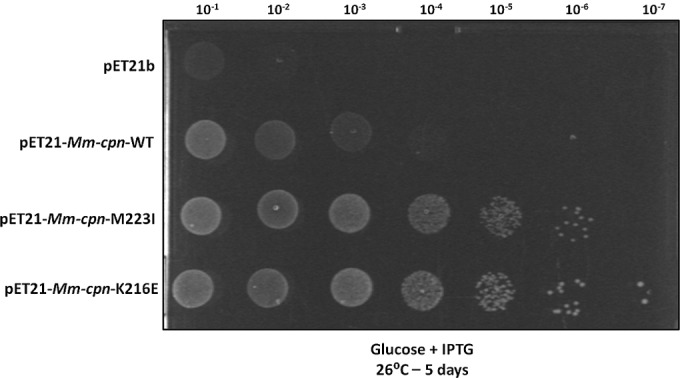
Random mutagenesis of Mm-cpn yielded mutants Mm-cpn-M223I and Mm-cpn-K216E with improved phenotype in E. coli under GroEL- and GroES-depleting conditions. TAB21 cells containing plasmids pET21, pET21-*Mm-cpn*, pET21-*Mm-cpn*-M223I, and pET21-*Mm-cpn*-K216E grown on 0.2% glucose and 1 mM IPTG at 30°C for 5 days.

Because Mm-cpn showed partial complementation TAB21 at reduced temperatures, we speculated that it should be possible to isolate variants with improved function in E. coli. To do this, we combined rounds of random mutagenesis with visual screening by growth under *groESL*-repressed conditions. pET-*Mm-cpn* was mutagenized by growth in the XL-1 mutator strain of E. coli, and TAB21 cells were transformed with a pool of plasmids prepared from this strain, with selection on glucose plates at 26°C, 30°C, and 37°C. Under these conditions, we observed some faster-growing colonies at 26°C and 30°C. After restreaking and verification of the phenotype, plasmids were isolated from these putative mutants, and the *cpn* genes within them were sequenced. Two single point mutations, one at position 216 from lysine to glutamate and the second at position 223 from methionine to isoleucine, were isolated using this procedure. To ensure that no other mutations could be causing the improved-growth phenotype, we individually reintroduced both M223I and K216E mutations into the *cpn* gene in pET-*Mm-cpn* and analyzed the ability of these mutants to support growth following dilution and spotting on glucose plates as described in Materials and Methods. Growth for both mutants was clearly faster than for wild-type (WT) Mm-cpn organisms under GroES/GroEL-limiting conditions (Fig. 2). We also examined the growth properties in liquid medium of the strains expressing the mutated Mm-Cpn proteins (see Fig. S1 posted at http://epapers.bham.ac.uk/2176/). Cells ceased to grow after ∼2 h of GroES/GroEL depletion unless they expressed Mm-cpn proteins. Cells expressing Mm-cpn showed slow and limited growth beyond this point, while those expressing Mm-cpn-K216E or Mm-cpn-M223I grew rapidly and to a high optical density, consistent with the phenotype seen on solid medium. To ensure that the phenotype was not due to improved expression or assembly, we compared the proteins on SDS-PAGE and native gels. All the Mm-cpn proteins were expressed at high levels when induced (see Fig. S2A posted at http://epapers.bham.ac.uk/2176/) and assembled equally well into ∼900-kDa complexes (see Fig. S2B posted at the same URL).

K216 and M223 are both in the apical domain of the Mm-cpn complex and attain different positions in its open and closed conformations (see Fig. S3 posted at http://epapers.bham.ac.uk/2176/). The K216 residue faces toward the inner surface of the cavity and contributes to the hydrophilic nature of the cavity, as described previously (38). The M223 residue lies in a loop beneath helix 11 near the helical protrusion and is on the outer surface of the chaperonin in a surface representation of the closed Mm-cpn complex. When compared across 20 different archaeal species, K216 and M223 were found to be 100% and 95% conserved, respectively.

### Mm-cpn containing mutations at K216 can replace GroEL function.

Although the results mentioned above showed that Mm-cpn had some ability to replace GroES and GroEL, which was further enhanced by the two mutations, they do not demonstrate complete functionality, as some residual group I activity may be present even in the depleted strains. We therefore investigated the ability of the different Mm-cpn variants to support growth in the complete absence of GroEL. This was done using strain AI90, which has a kanamycin resistance cassette replacing *groEL*. Growth of this strain is maintained by *ptrc*-regulated *groEL* on an expression plasmid that also encodes a *sacB* gene (32, 33). When a plasmid expressing Mm-cpn is introduced into this strain, cells will undergo plasmid shuffling, and those in which the original *groEL*-bearing plasmid has been displaced can be selected for by growth on sucrose (see Fig. S4 posted at http://epapers.bham.ac.uk/2176/); loss of the plasmid can be confirmed by chloramphenicol sensitivity of the colonies. Growth will occur only if the Mm-cpn variant can completely replace GroEL.

**FIG 3:**
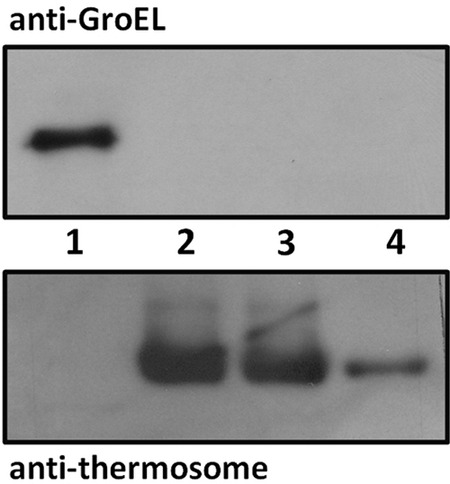
AI90 cells expressing Mm-cpn-K216E contain no GroEL protein. Western blots of soluble protein extracts from cultures using anti-GroEL and antithermosome antibodies. Lane 1, DH5α; lane 2, AI90/ptrc-*Mm-cpn*-K216E (26°C); lane 3, AI90/ptrc-*Mm-cpn*-K216E (30°C); lane 4, Mm-cpn purified protein (low concentration).

Cells were transformed with plasmids expressing Mm-cpn, Mm-cpn-M223I, and Mm-cpn-K216E and grown at 26°C, 30°C, and 37°C in the presence of sucrose. As a positive control, cells transformed with plasmid bearing wild-type *groEL* were also grown under similar conditions. Apart from the positive control, growth was observed only for cells expressing the mutant Mm-cpn-K216E. These cells showed distinct visible colonies after 3 days at 26°C and 30°C in numbers comparable to those observed for cells expressing GroEL (Table 1). Cells expressing Mm-cpn-K216E also formed visible colonies at 37°C after 5 days. Loss of the plasmid-encoded *groEL* from these transformants was confirmed by chloramphenicol sensitivity and by colony PCR (see Fig. S5 posted at http://epapers.bham.ac.uk/2176/). The transformants expressing Mm-cpn-K216E showed a complete absence of GroEL protein (Fig. 3, upper panel, lanes 2 and 3) and expression of Mm-cpn protein (Fig. 3, lower panel, lanes 2 and 3). Thus, the Mm-cpn-K216E mutant can support growth of cells completely lacking GroEL, but wild-type Mm-cpn and the Mm-cpn-M223I mutant cannot.

**TABLE 1:** Analysis of Mm-cpn functionality in Δ*groEL* strain AI90 incubated in LB containing sucrose and IPTG

Temp (°C)	No. of colonies^*a*^ for cells expressing:			
	GroEL	Mm-cpn WT	Mm-cpn M223I	Mm-cpn K216E
26	+++	−	−	++
30	+++	−	−	++
37	+++	−	−	+

a The results are indicative of the average number of colonies obtained for each sample from three independent experiments. The number of cells transformed, amounts of DNA used, and dilutions plated were uniformly maintained to be of equal number. Symbols: −, no colonies; +, between 10 and 50 colonies; ++, between 50 and 100 colonies; +++, between 100 and 200 colonies.

To further assess the significance of the K216 and M223 sites for improved GroEL complementation of Mm-cpn, we introduced a range of other amino acids at these positions. Those mutant proteins that expressed and assembled correctly were tested for complementation in TAB21. Cells expressing almost all K216 substitutions had an improved phenotype at 30°C compared to cells expressing Mm-cpn (Table 2; see also Fig. S6 posted at http://epapers.bham.ac.uk/2176/). Some substitutions (K216C, D, G, Q, S, and V) were better than others (K216A, F, L, P, T, and Y) and resulted in a K216E-like phenotype; the only mutant that led to poor growth was Mm-cpn-K216R. Every tested substitution at M223 uniformly displayed an M223I-like improved phenotype suggesting a broader range of functional flexibility at this residue than the residue K216 (Table 3). To validate the various phenotypes of TAB21 cells expressing different Mm-cpn-K216 substitutions, a selection of K216 mutants (K216A, D, L, Q, R, and S) were tested for their ability to sustain growth in complete absence of GroEL using the AI90 complementation system. AI90 cells expressing Mm-cpn-K216D, K216Q and K216S gave a number of colonies similar to the number of colonies of cells expressing Mm-cpn-K216E (Table 3). Cells expressing Mm-cpn-K216A and K216L showed poor growth, and expression of Mm-cpn-K216R did not support growth of the cells in complete absence of GroEL, consistent with the results from TAB21 analysis.

**TABLE 2:** Summary of the functional growth analysis of TAB21 cells expressing Mm-cpn-M223 and Mm-cpn-K216 mutants at 30°C^*a*^

Mutation in Mm-cpn	Complementation in TAB21 (30°C)
Lysine mutations	
K216A	++
K216C	+++
K216D	+++
K216F	++
K216G	+++
K216L	++
K216P	++
K216Q	+++
K216R	+
K216S	+++
K216T	++
K216V	+++
K216Y	++
Methionine mutations	
M223I	+++
M223F	+++
M223G	+++
M223E	+++
M223L	+++
M223R	+++
M223S	+++
M223V	+++
M223W	+++
M223Y	+++

a The results are indicative of the average number of colonies obtained for each sample from three independent experiments. The number of cells transformed, amounts of DNA used, and dilutions plated were uniformly maintained to be of equal number. Symbols: −, no colonies; +, between 10 and 50 colonies; ++, between 50 and 100 colonies; +++, between 100 and 200 colonies.

**TABLE 3:** Summary of the functional growth analysis of AI90 cells expressing Mm-cpn K216 mutants at 30°C and 37°C^*a*^

Addition(s) to LB agar	Temp (°C)	No. of colonies for cells expressing:						
		Mm-cpn K216E	Mm-cpn K216A	Mm-cpn K216D	Mm-cpn K216L	Mm-cpn K216Q	Mm-cpn K216R	Mm-cpn K216S
Sucrose	30	−	−	−	−	−	−	−
Sucrose and IPTG	30	++	+	++	+	++	−	++
Sucrose	37	−	−	−	−	−	−	−
Sucrose and IPTG	37	+	−	−	+	−	−	+

a The results are indicative of the average number of colonies obtained for each sample from three independent experiments. The number of cells transformed, amounts of DNA used, and dilutions plated were uniformly maintained to be of equal number. Symbols: −, no colonies; +, between 10 and 50 colonies; ++, between 50 and 100 colonies; +++, between 100 and 200 colonies.

### Complementation by Mm-cpn requires ATPase activity.

We wished to determine whether the complementing Mm-cpn mutants are functioning as true ATP-dependent chaperones when they complement loss of GroEL and GroES or whether complementation is due to some other indirect effect, such as providing a larger hydrophobic surface on which nascent unfolded proteins could bind, thus reducing the need for GroEL. To test this, the mutation D386A that blocks the ATPase activity of the chaperonin was introduced. The D386A mutant can bind to ATP but is unable to hydrolyze it (15, 48), hence stopping the chaperonin from undergoing a folding cycle by locking Mm-cpn in its ATP-bound open conformation. If Mm-cpn-K216E and Mm-cpn-M223I are functioning by simply binding to the clients while in an open conformation, the additional D386A mutation should not affect the phenotype of the mutants. If, on the other hand, the mutants are still acting as chaperonins requiring a full ATPase cycle, the additional D386A mutation should prevent Mm-cpn from supporting growth of the cells.

**FIG 4:**
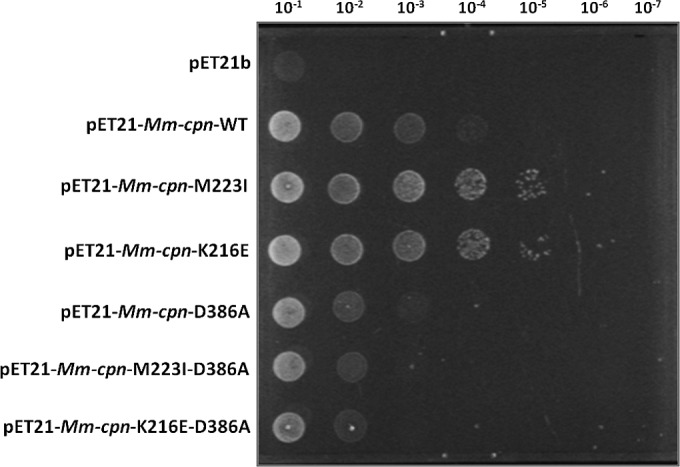
Growth of D386A mutants at 30°C under GroEL-depleting conditions. TAB21 cells expressing Mm-cpn-D386A mutants as indicated under pET21 vector grown on 0.2% glucose and 1 mM IPTG at 30°C for 5 days.

The D386A mutation was introduced into the *cpn* gene in pET-*Mm-cpn*, pET-*Mm-cpn*-K216E, and pET-*Mm-cpn*-M223I. Strains containing these plasmids were first checked to confirm that the Mm-cpn derivatives were correctly expressed and assembled in TAB21 (see Fig. S7 posted at http://epapers.bham.ac.uk/2176/) and then checked for complementation on glucose plates. As shown in Fig. 4, introduction of D386A into Mm-cpn significantly reduces its ability to complement for loss of GroES and GroEL, as do the Mm-cpn-K216E-D386A and Mm-cpn-M223I-D386A double mutants. Thus, loss of ATPase activity severely affects the complementing ability of the wild-type and mutant Mm-cpn proteins. This supports the hypothesis that the Mm-cpn-K216E and Mm-cpn-M223I mutants act as genuine chaperonins and must complete an ATP-dependent chaperonin cycle to function in E. coli.

### Biochemical characterization of Mm-cpn-M223I and Mm-cpn-K216E.

We analyzed the biochemical properties of the mutants to see whether a reason for their improved function in E. coli could be determined. We envisaged that the mutations could change the biochemical behavior of chaperonin oligomers to enable them to cycle more rapidly or to bind or fold client proteins more effectively. To test this, we purified the wild-type Mm-cpn and the Mm-cpn-K216E and Mm-cpn-M223I proteins (see Fig. S8 posted at http://epapers.bham.ac.uk/2176/). The melting temperatures as deduced from circular dichroism melting curves were similar to that of the wild-type protein (results not shown), showing that there are no significant differences in thermal stability between the proteins. We therefore tested for their ability to bind and fold clients, examined their rates of ATP hydrolysis, and compared their nucleotide-dependent conformational cycle to that of wild-type Mm-cpn.

**FIG 5:**
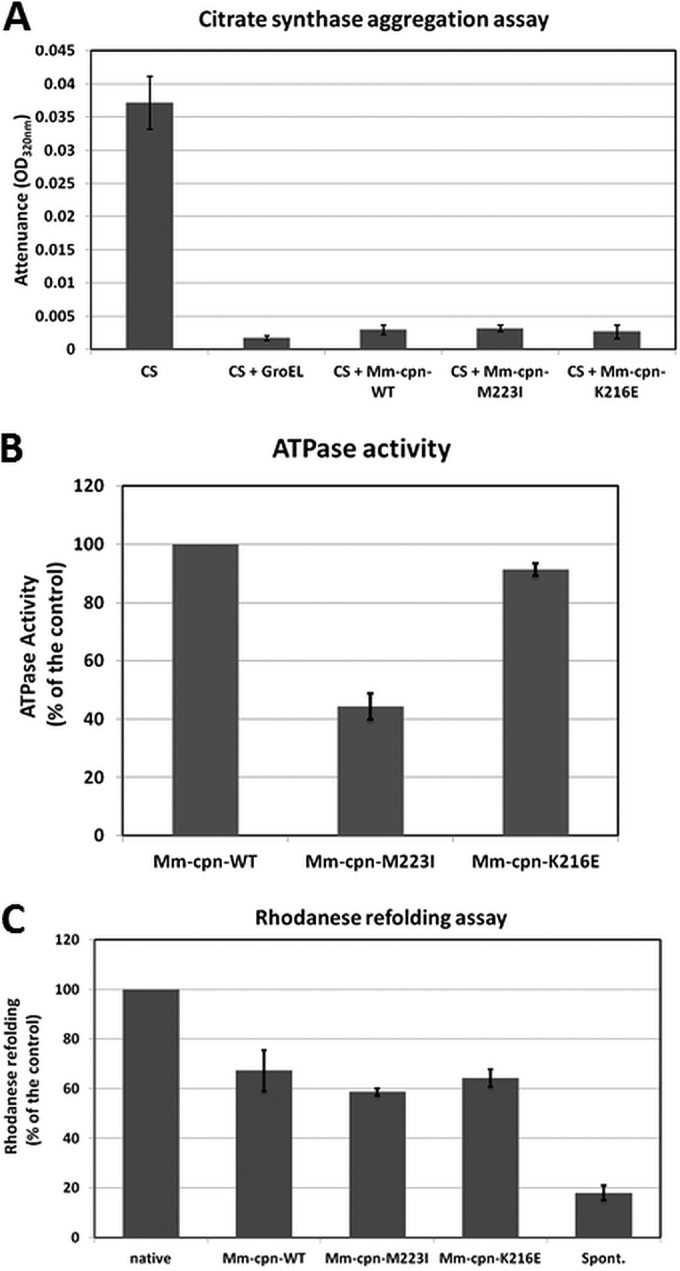
(A) Binding ability of Mm-cpn mutants as assessed by citrate synthase aggregation assay. Citrate synthase (CS) in the absence or presence of the various Mm-cpn proteins as indicated was incubated at 50°C, and the increase in attenuance was measured at 320 nm for 30 min as an endpoint for the assay. The Mm-cpn proteins and CS were used in equimolar concentrations (ratio of Mm-cpn complex to enzyme subunit). The data represent averages from three independent experiments. (B) ATP hydrolysis activity of Mm-cpn mutants. The Mm-cpn proteins were incubated in appropriate buffers at 37°C, and the ATPase activity was initiated by adding 1 mM ATP (final concentration) and continued for 15 min. The release of phosphate was assayed by the malachite green-molybdate method, and the absorbance at 640 nm was recorded. The data observed for Mm-cpn mutants are plotted as percentages of the data obtained for the wild type. (C) Rhodanese-refolding ability of Mm-cpn mutants. GuHCl-denatured rhodanese was incubated in the absence or presence of various proteins as indicated in an appropriate buffer containing 1 mM ATP at room temperature for 30 min. The data observed for Mm-cpn proteins are plotted as a percentage of the activity of native rhodanese.

We first tested the ability of Mm-cpn mutants to bind to porcine heart citrate synthase as a nonnative model substrate in an aggregation prevention assay as described previously (34). Incubation of citrate synthase at 50°C resulted in a rapid increase in OD_320_ of the solution over a period of 30 min. However, the presence of equimolar concentrations (Mm-cpn complex to enzyme subunit) of Mm-cpn, Mm-cpn-K216E, or Mm-cpn-M223I was sufficient to suppress the aggregation (Fig. 5A). Thus, the Mm-cpn mutant complexes can bind substrate protein efficiently and behave like WT Mm-cpn in this particular assay, but there is no clear difference in the functions of the mutant and wild-type proteins.

We assessed ATPase activities for all three proteins (Fig. 5B) and observed that the mutants can effectively hydrolyze ATP. The mutant Mm-cpn-K216E exhibited a rate of ATP hydrolysis fairly similar to that of the wild-type, whereas a reproducible drop in activity was observed for the mutant Mm-cpn-M223I.

Client refolding was assessed using rhodanese, which is widely used as a model client to test the *in vitro* refolding ability of both GroEL (39, 40) and Mm-cpn (34). Rhodanese was denatured using 6 M guanidinium chloride (GuHCl) and refolded by incubation with an equimolar amount of Mm-cpn wild-type or mutant proteins in the presence of ATP. As shown in Fig. 5C, approximately 60% to 70% recovery of native rhodanese was observed for Mm-cpn-M223I and K216E. There was not a significant difference in the values obtained for the mutants compared to those obtained for the wild type as observed here or reported previously. However, Mm-cpn-M223I consistently showed a slightly lower refolding activity than did Mm-cpn-K216E, which in turn showed activity that was almost identical to that of Mm-cpn.

**FIG 6:**
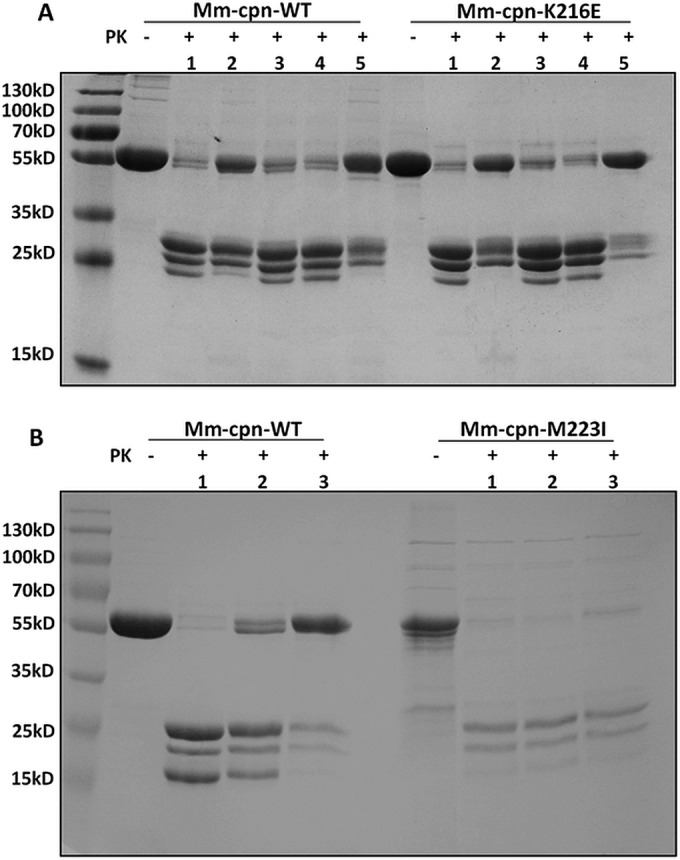
Protease sensitivity test for Mm-cpn-M223I and Mm-cpn-K216E. Protection against proteinase K (PK) for Mm-cpn, Mm-cpn-M223I, and Mm-cpn-K216E as observed by SDS-PAGE. (A) Analysis of Mm-cpn and Mm-cpn-K216E without (−) or with (+) PK in the absence of ATP (lane 1) or in the presence of ATP (lane 2), ADP (lane 3), AMP-PNP (lane 4), or ATP-AlF_x_ (lane 5), all at 1 mM. All gels were stained by Coomassie blue. (B) Analysis of Mm-cpn and Mm-cpn-M223I without (−) or with (+) PK in the absence of nucleotide (lane 1) or in the presence of ATP (lane 2) or ATP-AlF_x_ (lane 3).

Chaperonins exhibit different conformations at different stages of the folding cycle. To determine the asymmetric conformations of chaperonin complexes in the presence of different nucleotides, a simple assay based on susceptibility of digestion by a protease has been described (41, 42) and extensively used for both group I and group II chaperonins. If the point mutations Mm-cpn-K216E and Mm-cpn-M223I led to conformational changes of the complex or an alteration of time spent in the different conformations, the pattern of proteolytic digestion would be different from that of the wild type. To test this possibility, both mutants were subjected to a proteinase K sensitivity assay. Mm-cpn-K216E was partially protected against proteolytic cleavage in the presence of ATP and to a greater extent in the presence of ATP-AlFx, similar to the observations for wild-type protein (Fig. 6A). The mutant Mm-cpn-M223I, on the other hand, was rather weakly protected against protease digestion (Fig. 6B), suggesting that this protein spends more time in open conformation rather than closed, consistent with its lower ATPase activity. When the nonhydrolyzable ATP analogue, AMP-PNP, was used, effective proteinase K protection was reduced, consistent with ATP hydrolysis being important to attain the closed conformation as reported previously (15, 48) (Fig. 6A).

## DISCUSSION

We have demonstrated that a group II homo-oligomeric chaperonin from a mesophilic archaeon can partly replace E. coli GroEL and GroES function *in vivo* and that mutations that show improved function can be isolated and are sufficient in the case of mutations at K216 to allow deletion of the *groEL* gene. The biochemical endpoint assays reported here do not show any significant differences between the mutants and the wild-type Mm-cpn protein that might explain the improved ability of the mutants, in particular the K216 mutants, to complement for loss of GroEL function; more-detailed analysis may yet reveal subtle differences that are important *in vivo*. We also considered the possibility that better complementation by Mm-cpn would be obtained by coexpressing the prefoldins PfdA and PfdB from M. maripaludis, as these proteins are known to improve group II chaperonin function (43). Successful coexpression was achieved, but no improvement in complementation was observed under our experimental conditions (data not shown). We speculate that improved function is due to improved folding of a specific subset of clients, which is hence not captured by the assays that we used here. We note that K216 faces the inside of the chaperonin cage (see Fig. S3 posted at http://epapers.bham.ac.uk/2176/), so it is possible that the effect that we see with mutants at this position is in part due to a loss of positive charge on the cage surface. The wall of the GroEL cavity is enriched with negatively charged residues (net surface charge is −42) in contrast to the comparatively neutral distribution of the Mm-cpn cavity (19), and the negatively charged clusters of the GroEL cage wall have been implicated in facilitating the folding of bacterial clients (44, 45). Changing three negatively charged residues facing the inner cavity of GroEL to positive results in strong reduction of cell growth (44). This suggests the likelihood of negatively charged residues having a role in efficient folding by GroEL, potentially by providing local repulsion to acidic proteins, predominantly found in E. coli (46). Although the interpretation of these results has been challenged (47), it would be interesting to change the more positively charged residues facing the inner cavity of Mm-cpn to negative, ideally corresponding to the ones tested by Tang et al. (44) and to analyze the effects on E. coli growth in the absence of GroEL.

We also propose that the improved complementation of the mutant Mm-cpn-M223I despite its relatively lower ATPase activity could be due to better client binding owing to slower folding kinetics. Because it spends more time in an open conformation, a larger number of client proteins could bind and hence stabilize transiently. However, folding of at least some clients (even with slow ATP turnover) would depend on Mm-cpn-M223I, as blocking the ATPase activity by the D386A mutation leads to cessation of growth. The results thus suggest that both active folding of clients and slow aggregation kinetics of the misfolded proteins could jointly contribute to the improved GroEL-complementing ability of Mm-cpn-M223I.

Our findings provide an insight into the relationship between the group I and group II chaperonins, showing that they are not as diverse in their functional properties as has been previously suggested, and additionally provide a novel experimental platform that can be used to rapidly analyze the *in vivo* properties of a group II chaperonin.
